# Challenges Assessment in Endodontics Among Undergraduate Students

**DOI:** 10.7759/cureus.43215

**Published:** 2023-08-09

**Authors:** Mishal Almutairi, Mustafa Hussein Alattas, Ahmed Alamoudi, Sarah Ahmed Bahammam, Bassam Zidane, Nawaf Almutairi, Hammam A Bahammam

**Affiliations:** 1 College of Dentistry, Qassim University, Buraidah, SAU; 2 Conservative Dental Science and Endodontics, Qassim University, Buraydah, SAU; 3 Oral Biology, King Abdulaziz University, Jeddah, SAU; 4 Pediatric Dentistry and Orthodontics, Taibah University, Taibah, SAU; 5 Restorative Dentistry, King Abdulaziz University, Jeddah, SAU; 6 Conservative Dental Science and Endodontics, Qassim University, Buraidah, SAU; 7 Pediatric Dentistry, King Abdulaziz University, Jeddah, SAU

**Keywords:** dentistry, dental education, medical education assessment, health education & awareness, endodontic

## Abstract

Objective

To explore perceptions of undergraduate dental students regarding difficulties faced during endodontic procedures.

Methods

An e-questionnaire was sent to 57 fourth-year and 45 fifth-year dental students. It comprised questions regarding demographic data and difficulties faced during different steps of the endodontic treatment. The responses were recorded on a 5-point Likert scale. During dichotomization, responses where one and two were chosen were considered yes whereas responses where three, four, and five were chosen were considered no. The sample size (SS) calculated using the Qualtrics SS calculator was 89. Data were analyzed using SPSS version 20. Frequencies and percentages were calculated. A p-value of less than 0.05 was considered significant. The chi-square test was applied for comparison based on the students’ academic year and genders.

Results

Ninety students responded with an 88.2% response rate. Most students reported not facing any difficulty in differentiating healthy pulp and periapex from conditions of pulp pathosis (78.9%) and periapical pathosis (75.6%). Most males found access opening and de-roofing of the pulp chamber (35.6%) and working length determination (31.1%) difficult. Females reported having difficulty mostly in mesial and distal shift radiograph techniques (55.6%) and access openings (51.1%). No statistical difference was found based on an academic year or gender except in the use of mesial and distal shift techniques for radiograph acquisition

Conclusion

Assessing difficulties encountered by students during endodontic therapy can aid in the development of teaching strategies for preclinical and clinical instruction. Mesial and distal shift techniques, access cavity preparation, and working length determination are areas requiring more focus in the training process.

## Introduction

Endodontic treatment, commonly known as root canal therapy, is a vital aspect of modern dentistry. It plays a crucial role in preserving natural teeth, preventing the spread of infection, and maintaining oral health. The prerequisites for successful endodontic treatment comprise a thorough understanding of the normal and variant root canal anatomy [1], the ability to interpret radiographs and other diagnostic images, sound clinical decision-making skills, diagnosis of pulpal and periapical diseases, appropriate treatment planning and proper execution of each step of the root canal therapy including isolation, administration of anesthesia, access cavity preparation, working length determination, canal shaping and disinfection, obturation and placement of the coronal restoration [2,3]. An error occurring at any point in these steps can result in endodontic treatment failure [4,5]. Therefore, dental education must train future dentists in such a way that they become proficient in performing all these procedures independently and confidently [6]. Numerous studies have reported that the greater the dental student’s exposure to endodontic clinical cases during years of dental education, the better equipped he or she will be to practice endodontics as a professional in the coming years. Others have found that the quality of the completed endodontic treatment is a more important parameter, in assessing whether a student has attained enough proficiency, compared to the number of endodontic treatments performed [7-9].

Owing to the morphological complexity and diversity of the root canal system, the gravity of patient care, the intricacy of the endodontic treatment procedure, and the student's lack of confidence many dental students find endodontics to be a stressful, challenging, and complex subject to learn [10,11]. The majority of dental students believe that molar teeth, especially maxillary molars, are the most difficult to treat [12]. Ironically, the teeth that most often require endodontic treatment are the molars [13]. The perception of treatment difficulty among dental students significantly influences their confidence levels, motivation, and overall performance during endodontic procedures. These perceptions can be shaped by various factors, including the level of education, teaching methodologies, exposure to clinical cases, previous experiences, and individual learning styles [9]. The successful execution of endodontic procedures requires a combination of technical skills, theoretical knowledge, and clinical experience. As dental students embark on their journey to become competent practitioners, understanding their perceptions of the complexity and difficulty associated with endodontic treatment becomes imperative. Students’ perceptions can offer insightful ideas and criticism for improving the curriculum and the learning environment [14]. The process of assessment and evaluation is fundamentally based on feedback, and students’ feedback can yield crucial data that can help the student and the course succeeds by improving teaching and enhancing learning [11]. Understanding the perceived difficulties of undergraduate dentistry students can shed light on potential areas of improvement within the dental curriculum. Educators can utilize this knowledge to develop comprehensive and effective instructional methods, tailored to the specific educational needs of their students, such as hands-on clinical training, simulation exercises, and interactive learning approaches. They can also lend targeted support to enhance confidence and skills [6]. By addressing these difficulties early in the educational process, dental schools can better equip their students to handle the complexities of endodontic treatment, thereby enhancing patient care and promoting overall oral health. This cross-sectional survey aimed to add to the existing literature by exploring the perceptions of undergraduate dental students regarding difficulties faced during endodontic procedures. The findings of this study can help develop potential strategies to overcome these difficulties and assist educators in shaping dental curricula, teaching methodologies, and support systems to better prepare undergraduate dental students for successful endodontic practice.

## Materials and methods

This cross-sectional survey was conducted at Qassim University Dental School where the undergraduate competency-based dental curriculum is taught over a period of five years. The teaching of endodontics begins in the third year of dental school and continues till the fifth year. It includes both imparting theoretical knowledge and teaching practical skills. The third-year dental students receive pre-clinical teaching which comprises didactic lectures and a preclinical endodontic skills course. This course entails 60 hours of practical instruction in the skills lab to teach dental students how to perform root canal procedures using both manual and rotary instrumentation methods. They perform these procedures on extracted teeth as well as acrylic teeth mounted on phantom heads in the skills lab. Training in clinics starts in the first term of their fourth year of undergraduate dental school. Progressively increasing the complexity of the knowledge and procedures helps reduce cognitive load and enhances learning [15]. That is why in the fourth year of undergraduate dental school, students are expected to perform root canal treatment on teeth with less complex anatomy such as incisors/premolars, and in the fifth year on teeth with more complex morphology such as molars. It is mandatory for students to complete a certain number of root canal procedures each year. These pre-clinical and clinical procedures are carried out under the supervision of endodontists as an imperative part of the patient care block.

The sample population for this questionnaire-based cross-sectional survey included fourth and fifth-year undergraduate students enrolled at the Qassim University Dental School. All the study participants had engaged in endodontic, laboratory, and clinical, procedures during their third, fourth, and fifth years of dental school. The institutional review board at the Qassim University School of Dentistry provided ethical approval (registration number #EA/6123/2021) for this study. The questionnaire used was adopted from a study conducted by Tavares et al. [6]. It comprised two parts. Part one collected demographic data (academic year/ gender), while Part two included 10 questions regarding the different steps of the endodontic treatment and inquired whether the students found these steps difficult. The response to each question was recorded on a five-point Likert scale (LS) as follows: one (totally agree); two (agree); three (neutral); four (disagree); five (totally disagree). Subsequently, the data were dichotomized for ease of interpretation. During dichotomization, responses one and two on the LS were considered yes and responses three, four, and five on LS were considered no. The sample size (SS) was calculated using the Qualtrics SS calculator. The cumulative target student population was 102. With a 99% confidence level and a 5% error margin, 89 was calculated as an acceptable SS. At the end of the academic year, 57 fourth-year dental students and 45 fifth-year dental students received the survey e-questionnaire via Google form link through the official university email. There were two emails sent out as reminders, at one-week intervals of sending the e-questionnaire link to the potential participants. The students were informed that their responses will be anonymous. They were also told that participation in the survey was entirely voluntary, and they were allowed to refuse participation or withdraw at any time without specifying any reason. Only the respondents were included in the study. Non-respondents were excluded. Data were analyzed using SPSS version 20.0 for Windows (IBM corp., Armonk, NY, USA). Frequencies and percentages were calculated. A p-value of less than 0.05 was considered significant. The chi-square test was applied for comparison based on the student’s academic year and their genders.

## Results

**Figure 1:**
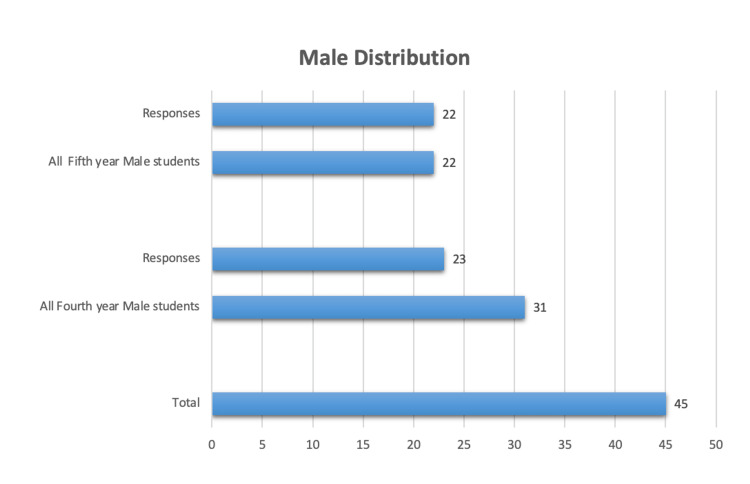
Male Distribution

**Figure 2:**
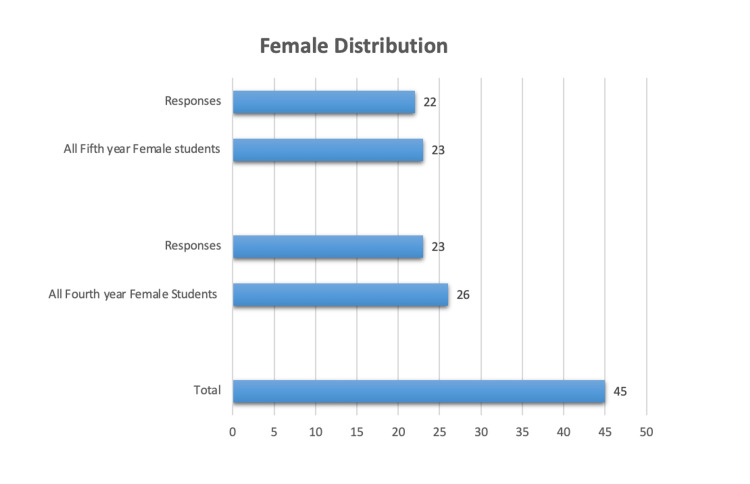
Female Distribution

Out of the 102 dental students who received the survey e-questionnaire, 53 were males (31 fourth-year and 22 fifth-year dental students) and 49 were females (26 fourth-year and 23 fifth-year students). A total of 90 students responded resulting in an 88.2% response rate. The number of male and female respondents was equal. The distribution of male and female study participants, based on their academic year and responsiveness, is demonstrated in Figures 1, 2 respectively.

About 78.9% of the participants reported that they have the knowledge required to distinguish between healthy pulp and different conditions of pulp pathosis (Table 1, question 1). Most students (75.6%) believed they know how to differentiate between healthy periapical tissues and different conditions of periapical tissue pathosis (Table 1, question 2). Around 71.1% of all fourth and fifth-year dental students reported not having any difficulties in performing adequate anesthesia whereas 28.9% admitted to facing difficulties in administrating anesthesia effectively (Table 1, question 3). Around 37% of fourth-year students reported having difficulties with rubber dam application compared to only 22.7% of fifth-year students (Table 1, question 4). The percentages of fourth and fifth-year students that reported experiencing difficulties in taking radiographs using the mesial and distal shift techniques are 47.8% and 36.4% respectively (Table 1, question 5). Similarly, 43.3% of students reported having difficulty accessing cavity preparation and pulp chamber de-roofing (Table 1, question 6). A large proportion of participants (36.7%) responded that they find working length determination difficult (Table 1, question 7). The percentages of students that reported not facing any difficulties during canal instrumentation, intracanal medicament placement, and obturation are 68.9%, 65.6%, and 73.3% respectively (Table 1, questions 8,9,10). There was no statistical difference in the undergraduate dental students’ self-perceived difficulties in relation to endodontic treatment based on the academic year. 

**Table 1:** Undergraduate students’ self-perceived difficulties in relation to endodontic treatment on the basis of the academic year *Chi-square test

Question Statements	Students Responses Academic year wise n (%)						P-Value^*^
	Fourth Year (n=46)		Fifth year (n=44)		Total Responses (n=90)		
	Yes	No	Yes	No	Yes	No	
I have the knowledge to differentiate between healthy pulp and different conditions of pulp pathosis.	37 (80.4)	9 (19.6)	34 (77.3)	10 (22.7)	71 (78.9)	19 (21.1)	0.71
I have the knowledge to differentiate between healthy periapical tissue and different conditions of periapical tissue pathosis.	34 (73.9)	12 (26.1)	34 (77.3)	10 (22.7)	68 (75.6)	22 (24.4)	0.71
I don't have any kind of difficulties performing adequate anesthesia.	32 (69.6)	14 (30.4)	32 (72.7)	12 (27.3)	64 (71.1)	26 (28.9)	0.74
I don't have any kind of difficulties with the rubber dam application.	29 (63)	17 (37)	34 (77.3)	10 (22.7)	63 (70)	27 (30)	0.14
I don't have any kind of difficulties with the mesial or distal shift technique.	24 (52.2)	22 (47.8)	28 (63.6)	38 (36.4)	52 (57.8)	38 (42.2)	0.27
I don't have any kind of difficulties while doing the access opening and de-roofing the pulp chamber.	25 (54.3)	21 (45.7)	26 (59.1)	18 (40.9)	51 (56.7)	39 (43.3)	0.65
I don't have any kind of difficulties to determine the working length.	30 (65.2)	16 (34.8)	27 (61.4)	17 (38.6)	57 (63.3)	33 (36.7)	0.71
I don't have any kind of difficulties in root canal instrumentation and flaring.	28 (60.9)	18 (39.1)	34 (77.3)	10 (22.7)	62 (68.9)	28 (31.1)	0.09
I don't have any difficulties in the placement of intra-canal medication.	30 (65.2)	16 (34.8)	29 (65.9)	15 (34.1)	59 (65.6)	31 (34.4)	0.95
I don't have any difficulties in obturation of the root canal.	37 (80.4)	9 (19.6)	29 (65.9)	15 (34.1)	66 (73.3)	24 (26.7)	0.12

In this study, the difficulties reported by dental students in performing endodontic treatment were also compared between genders (Table 2). Most male and female students reported having the least difficulty differentiating between healthy pulp and conditions of pulp pathosis (78.9%) and between healthy periapical tissues and conditions of periapical pathosis. (75.6%) The steps of endodontic treatment that most males found difficult were access opening and de-roofing of the pulp chamber (35.6%) followed by working length determination (31.1%) whereas female students reported encountering difficulty mostly during mesial and distal shift radiograph techniques (55.6%) and access opening (51.1%). No statistical difference was found between the two genders regarding endodontic treatment difficulties except in the use of mesial and distal shift techniques for radiograph acquisition. 55.6% of female students reported difficulty using the mesial and distal shift techniques compared to only 28.9% of males (Table 2, question 5).

**Table 2:** Undergraduate students’ self-perceived difficulties in relation to endodontic treatment on the basis of gender *Chi-square test

Question Statements	Students Responses Gender wise n (%)						P-Value^*^
	Male (n=45)		Female (n=45)		Total Responses (n=90)		
	Yes	No	Yes	No	Yes	No	
I have the knowledge to differentiate between healthy pulp and different conditions of pulp pathosis.	38 (84.4)	7 (15.6)	33 (73.3)	12 (26.7)	71 (78.9)	19 (21.1)	0.20
I have the knowledge to differentiate between healthy periapical tissue and different conditions of periapical tissue pathosis.	37 (82.2)	8 (17.8)	31 (68.9)	14 (31.1)	68 (75.6)	22 (24.4)	0.14
I don't have any kind of difficulties to perform an adequate anesthesia.	35 (77.8)	10 (22.2)	29 (64.4)	16 (35.6)	64 (71.1)	26 (28.9)	0.16
I don't have any kind of difficulties in the rubber dam application.	35 (77.8)	10 (22.2)	28 (62.2)	17 (37.8)	63 (70)	27 (30)	0.12
I don't have any kind of difficulties in the mesial or distal shift technique.	32 (71.1)	13 (28.9)	20 (44.4)	25 (55.6)	52 (57.8)	38 (42.2)	0.01
I don't have any kind of difficulties while doing the access opening and de-roofing the pulp chamber.	29 (64.4)	16 (35.6)	22 (48.9)	23 (51.1)	51 (56.7)	39 (43.3)	0.14
I don't have any kind of difficulties to determine the working length.	31 (68.9)	14 (31.1)	26 (53.8)	19 (42.2)	57 (63.3)	33 (36.7)	0.27
I don't have any kind of difficulties in root canal instrumentation and flaring.	35 (77.8)	10 (22.2)	27 (60)	18 (40)	62 (68.9)	28 (31.1)	0.07
I don't have any difficulties in placement of intra-canal medication.	33 (73.3)	12 (26.7)	26 (53.8)	19 (42.2)	59 (65.6)	31 (34.4)	0.12
I don't have any difficulties in obturation of the root canal.	36 (80)	9 (20)	30 (66.7)	15 (33.3)	66 (73.3)	24 (26.7)	0.15

## Discussion

The evolution of dental education has been a fascinating journey that has seen tremendous breakthroughs and significant advancements over time [6]. Traditionally, teaching methodologies have been passive and teacher-centered, consisting mainly of didactic lectures and rote memorization of information. The ability of a student to reproduce information shared by the teacher is the measure of competence in a teacher-centered curriculum. Even though the traditional methodologies have their merits, they leave little to no room for the development of critical thinking and decision-making skills in the students [16]. As a result, theoretical knowledge cannot be put to practical use, resulting in under-confident dentists who do not know how to apply their knowledge to everyday clinical scenarios. Due to these shortcomings, educationists are moving towards a more student-centered approach. The student holds prime importance in a student-centered curriculum. Under the guidance of a facilitator/tutor, students have the freedom to determine the order and pace of their learning, choose their own learning outcomes, select the best resources to help them accomplish their learning objectives, and evaluate their own progress. This kind of approach creates a sense of responsibility among the students regarding their learning [17]. Modern teaching methodologies focus on learning rather than teaching and aim to promote active learning based on the principles of constructivism [18,19]. Another important change that has occurred in dental education over time is the horizontal and vertical integration of different disciplines, allowing the correlation of information and the ability to relate theory to practice [20]. According to the theory of multiple intelligences proposed by Howard Gardner, there are numerous forms of intelligence and different people have different intellectual and learning abilities for example some can retain visual information better while others can learn more through verbal instructions [21,22]. Instructional methods for active learning include small group discussions, flipped classrooms, simulation, peer-assisted and blended learning. Small group discussions include tutorials, seminars, free group discussions, problem-based learning (PBL), case-based learning (CBL), task-based learning, and team-based learning [23]. No single instructional method can be declared the best. A combination of different teaching methods is considered most effective because different students learn in different ways [6]. These improvements in dental education have been possible because of the identification of shortcomings and limitations of traditional teaching strategies. Learning is an ongoing process and regular feedback fosters a culture of continuous improvement [24].

Even small errors can have significant consequences for patients in a clinical setting. It is of utmost importance for patient safety that students recognize and correct mistakes early on and practice procedures repetitively to achieve proficiency so that potential harm in real patient care scenarios can be prevented. The teacher must ascertain which step of the endodontic treatment the dental students are struggling with. What may be perceived as challenging by one student may be considered easy by another and vice versa. Knowing which step the students find difficult prompts the teacher to concentrate more on that step in order to solve the problem and guide the students to effectively perform root canal therapy [11].

The present study demonstrated that the step of endodontic treatment that most students found least difficult was the diagnosis. Most of the male and female students believed that they do not have any difficulty in differentiating healthy pulpal and periapical tissues from conditions of pulpal and periapical diseases. Some researchers that have evaluated the confidence levels of students regarding different endodontic treatment procedures have also reported a high level of confidence in students pertaining to appropriate pulpal and periapical diagnosis [9,25]. This may be attributed to the fact that in most dental schools, during the third year, students attend endodontic pre-clinical skills labs and lectures. In contrast, a study conducted in a Brazilian dental school reported that most dental students have difficulty diagnosing conditions of pulpal and periapical diseases. This may be ascribed to their traditional curriculum which does not integrate theoretical and clinical sciences and leads to fragmentation of knowledge [14].

Around 70% of students reported not having any difficulty in administrating anesthesia. This corresponds to the results of similar studies conducted by Kaplan et al. and Almohaimede [11,25]. Most of the students consider anesthesia administration relatively less difficult than other steps of root canal treatment probably because it is a procedure that is not exclusive to endodontic treatment. Students learn and administer anesthesia for many different treatment procedures during their clinical rotations in different departments e.g., for tooth extraction in oral surgery clinics. A similar perception among students was found regarding rubber dam application, with 70% reporting not facing any difficulty. Previous studies have also yielded similar results. Tavares et al. reported that around 68-90% of students did not perceive rubber dam application as difficult [6]. This may be because students receive extensive hands-on training in rubber dam application during their second and third years of study. In the current study, when inquired about instrumentation and canal flaring, 31.1% of dental students reported finding this step difficult. Regarding the obturation of root canals, 73.3% of students did not perceive having any difficulty. Different studies have yielded different reports regarding the perception of the difficulty of canal instrumentation and obturation among students [6,11,14,25]. About 34.4% of dental students in our study reported having difficulty with intracanal medicament placement. This differs from the results obtained by Tavares et al. who reported that around 35-67% of students encounter difficulty with the use of intracanal medicament [6]. These variations in the results of various studies may be attributed to the differences in the types of curricula, teaching strategies, instructional methods, clinical and pre-clinical exercises, use of simulation, and student-teacher interaction among the different dental schools where these studies were conducted. 

In the present study, students reported having the most difficulty with mesial and distal shift radiograph technique, access cavity preparation and pulp chamber de-roofing, and working length determination. These results are in agreement with those obtained from other studies [6,11,12,14]. These procedures are inherently complex and therefore require extensive theoretical knowledge and practical expertise. According to French research, preparing access cavities ranked third when students were asked which stage of root canal therapy, they were most apprehensive about. The reason cited for this was the extremely variable internal anatomy of teeth [26]. The measures that must be taken to reduce the difficulty faced by students during these procedures include integration of theory with clinical practice, demonstrations using practical models, use of apex locators, use of film holders for radiograph acquisition, use of appropriate magnification and illumination, exercises on artificial and extracted teeth, patient exposure and clinical case discussions [11,12,27].

Another interesting finding of our study is that only 19.4% of fourth-year dental students reported finding obturation/root filling difficult compared to 34.1% of fifth-year students. A study conducted by Murray et al. reported that when fifth-year students compared their endodontic treatment experiences with those in their fourth year of dental school, 40.6% found them to be more difficult [12]. The reason for this may be the increase in the morphological complexity of teeth to be treated. In the fourth year, dental students treat teeth with straight and single canals, whereas in the fifth-year students perform endodontic procedures on multi-rooted teeth with curved roots and canals. Overall, male students reported facing less difficulty in most procedures compared to female students. Females had the most difficulty in mesial and distal shift techniques, and males had the most difficulty in access cavity preparation and de-roofing followed by working length determination. Studies that have explored the self-confidence of students in performing different endodontic treatment steps have also demonstrated that male students are overall more confident about their treatment skills and abilities compared to their female counterparts [9]. Support and encouragement from teachers and good teacher-student communication may help female students overcome this problem.

One of the limitations of the current study is that the data was obtained from one dental school in one geographical location, therefore the results cannot be generalized. Further research on a larger scale should be conducted so that the difficulties faced by students may be identified and an assessment can be made regarding the best approach to reduce these difficulties. Feedback from the students is crucial for educational reforms and should be obtained periodically to improve learning.

## Conclusions

Assessing the challenges and difficulties encountered by students during root canal therapy can aid in the development of teaching strategies for preclinical and clinical instruction. According to our findings, the use of mesial and distal shift radiographic techniques, access cavity preparation, and working length determination are areas that need more focus in the training process. Feedback encourages students to reflect on their clinical experiences and allows teachers to tailor instructions to individual student's needs and learning styles. This personalized approach ensures that students receive guidance and support that best suits their unique development requirements.
